# Development of an EORTC Quality of Life Module for Renal Cell Cancer Patients: Phase I

**DOI:** 10.1192/j.eurpsy.2022.1952

**Published:** 2022-09-01

**Authors:** L. Wintner, E. Beisland, C. Beisland, M. Van Hemelrijck, B. Holzner

**Affiliations:** 1 Medical University of Innsbruck, Department Of Psychiatry, Psychotherapy And Psychosomatics, Innsbruck, Austria; 2 Western Norway University of Applied Sciences, Department Of Health And Social Sciences, Bergen, Norway; 3 Haukeland University Hospital, Department Of Urology, Bergen, Norway; 4 King’s College London, Faculty Of Life Sciences And Medicine, Translational Oncology And Urology Research (tour), London, United Kingdom

**Keywords:** Quality of Life, renal cell cancer, questionnaire module development, patient-reported outcomes

## Abstract

**Introduction:**

In light of rising incidence rates and a mostly late diagnosis, renal cell cancer (RCC) patients are heavily burdened by both their disease and treatment. The structured assessment of their quality of life using patient-reported outcome (PRO) measures is important in order to provide them with appropriate interventions to maintain or improve their quality of life. Available questionnaires are predominantly symptom indices or were developed without conducting patient interviews.

**Objectives:**

Hence, we report on the ongoing phase I development of an EORTC module for RCC patients, which will be used together with the EORTC QLQ-C30 core questionnaire.

**Methods:**

Following the EORTC Quality of Life Group’s Module Development Guidelines, a systematic literature review was conducted. Based on this review, issues were extracted and presented to healthcare professionals (HCPs) and patients for relevance assessment.

**Results:**

133 publications (14 on the development of RCC-specific PRO measures, 3 qualitative studies, 37 randomised controlled trials, 79 quantitative studies) were identified from which 150 unique issues were extracted. The issue list was reviewed by 14 HCPs (8 clinicians, 3 nurses, 2 psychooncologists, 1 physiotherapist) from 3 countries (Austria, Norway, United Kingdom) and rated regarding their relevance. An additional 13 issues were mentioned in the HCP interviews and included in the issue list.

**Conclusions:**

The extended list of issues is currently used to interview patients. Data collection is expected to be completed by the conference, thus the poster will present the combined relevance scores (HCPs and patients) and the issues selected for the preliminary module to be tested in phase 3.

**Disclosure:**

This study is funded by the EORTC Quality of Life Group (Grant 007/2019).

